# Lenalidomide treatment of Japanese patients with myelodysplastic syndromes with 5q deletion: a post-marketing surveillance study

**DOI:** 10.1007/s12185-023-03634-7

**Published:** 2023-07-26

**Authors:** Shuji Uno, Yoko Motegi, Kenichi Minehata, Yasuo Aoki

**Affiliations:** 1 Japan Medical-Hematology, Bristol-Myers Squibb K.K., Otemachi One Tower, 1-2-1 Otemachi, Chiyoda-Ku, Tokyo, 100-0004 Japan; 2 PMS Operations, Worldwide Patient Safety Japan, Bristol-Myers Squibb K.K., Tokyo, Japan

**Keywords:** Lenalidomide, Myelodysplastic syndromes with 5q deletion, Post-marketing surveillance study

## Abstract

Lenalidomide was approved in Japan for the treatment of patients with myelodysplastic syndromes associated with a 5q deletion (del 5q-MDS) in August 2010. A post-marketing surveillance (PMS) study enrolled 173 patients with del 5q-MDS who started lenalidomide treatment between August 2010 and September 2011 (mean ± standard deviation [SD] age 72.4 ± 9.0 years) and observed for up to 6 cycles or 6 months. Adverse drug reactions (ADRs) and serious ADRs were reported in 78.0% and 50.9% of patients. The most commonly observed ADRs were thrombocytopenia or platelet count decreased (46.2%), neutropenia or neutrophil count decreased (42.2%), and rash (23.1%). Of 114 patients who were red blood cell transfusion-dependent at baseline, 39 (34.2%) achieved transfusion independence during lenalidomide treatment. Of 173 patients, 19 (11.0%) had confirmed acute myeloid leukemia (AML) progression during the study. Moreover, long-term follow-up (3 years) was available for 68 of the 173 patients, of whom 12 (17.6%) progressed to AML during the additional period. This PMS study investigated the safety and effectiveness of lenalidomide in patients with del 5q-MDS. No new safety concerns were noted in routine clinical use in Japan and no evidence was found for an increased risk of AML progression following lenalidomide treatment.

**Supplementary Information:**

The online version contains supplementary material available at 10.1007/s12185-023-03634-7.

## Introduction

Myelodysplastic syndromes (MDS) are the term used to describe a group of acquired hematologic malignancies that are characterized by single or multiple lineage cytopenia, morphologic dysplasia, and ineffective hematopoiesis, and has the potential for acute leukemic transformation [1]. Anemia is the most common clinical manifestation of MDS, appearing in 80–85% of patients at diagnosis and negatively impacting the patient’s quality of life (QoL) [2]. In Japan, the incidence of MDS increases with age, particularly in individuals aged ≥ 70 years; the estimated median age at diagnosis is 76 years [3]. In 2008, the incidence of MDS in Japan was 3.8 per 100,000 men and 2.4 per 100,000 women [3].

According to the 1982 French–American–British (FAB) method of classification, there are five types of MDS: refractory anemia (RA), RA with ring sideroblasts (RARS), RA with excess of blasts (RAEB), chronic myelomonocytic leukemia, and RAEB in transformation [4]. In addition to FAB classification, MDS may also be categorized according to the World Health Organization (WHO) 2008 classification [5] and its 2016 revision [6]. The WHO 2008 classification includes seven types of MDS: refractory cytopenia (RC) with unilineage dysplasia, RARS, RC with multilineage dysplasia, RAEB-1 (< 5% blasts in the bone marrow, but 2–4% blasts in the blood; no Auer rods), RAEB-2 (< 5% blasts in the blood if Auer rods are present, 10–19% blasts in the bone marrow, or both), MDS-unclassified, and MDS associated with isolated deletion 5q (del 5q) cytogenic abnormality [5].

Given the heterogeneous nature of MDS, diagnosis and disease classification alone are often insufficient when selecting the best treatment strategy. Treatment of MDS is commonly guided by risk assessment according to the International Prognostic Scoring System (IPSS), which was developed in 1997 [7] and revised in 2012 [8]. The 1997 IPSS classifies patients with MDS into four risk categories (low, intermediate [int]-1, int-2, and high) based on the percentage of blasts, number of cytopenic lineages, and the presence of cytogenic abnormalities [7]. In patients with low-risk MDS (IPSS low or int-1), the main goals of treatment are improving cytopenia(s), reducing the burden of red blood cell (RBC) transfusion, and improving the patient’s QoL [9]. In patients with high-risk MDS (IPSS int-2 or high), treatment is primarily aimed at modifying the disease course, preventing disease progression, and increasing the chances of survival [9].

Treatment of low-risk MDS includes immunosuppressant therapy with anti-thymocyte globulin (± cyclosporine) to restore hematopoiesis, as well as erythropoiesis-stimulating agents (ESAs; ± granulocyte colony-stimulating factor) to correct MDS-related anemia [9]. However, ESA therapy is generally limited to patients with low endogenous erythropoietin levels (< 500 IU/L) and a low RBC transfusion burden (< 4 U every 8 weeks) [10]. In addition, patients who require regular RBC transfusions may develop iron overload and, therefore, require concomitant iron chelator therapy (e.g., deferasirox), which further increases the treatment burden [9].

Lenalidomide is an immunomodulatory agent that was approved in Japan for the treatment of patients with MDS associated with a del 5q cytogenic abnormality in August 2010 [11]. This approval was based on the findings of the phase 3 MDS-004 [12] and phase 2 MDS-007 [13] clinical trials in patients with low or int-1 risk MDS with del 5q. In the MDS-004 trial of RBC transfusion-dependent patients with MDS with del 5q (*n* = 139), lenalidomide provided a significantly greater erythroid response rate (proportion of patients with RBC transfusion independence for ≥ 26 weeks) at 5 mg/day (20/47; 42.6%) and 10 mg/day (23/41; 56.1%) compared with placebo (3/51; 5.9%) [both doses *P* < 0.001 vs. placebo]; the median increase in hemoglobin levels from baseline with lenalidomide 5 and 10 mg/day was 5.2 and 6.3 mg/dL, respectively [12]. In the MDS-007 trial of 11 Japanese patients with del 5q-MDS, all five patients with transfusion-dependent MDS achieved RBC transfusion independence with lenalidomide 10 mg/day, and hemoglobin levels increased by a median of 6.0 g/dL across all patients [13].

Because the number of Japanese patients treated with lenalidomide in the MDS-007 trial was very low (*n* = 11) [13], a post-marketing surveillance (PMS) study was conducted in all patients treated with lenalidomide in Japan. The aim of this analysis was to evaluate the safety and effectiveness of lenalidomide in patients with MDS with del 5q and to assess the rate of transition from del 5q-MDS to acute myeloid leukemia (AML) in patients who had received lenalidomide.

## Materials and methods

### Study design

This was a prospective, non-interventional, all-case PMS study of lenalidomide in Japan. In this study, case report forms (CRFs) were collected from all eligible individuals who started treatment with lenalidomide in the regimen approved for its use in Japan (i.e., for del 5q-MDS or relapsed or refractory multiple myeloma [RRMM]). In this analysis, the safety and effectiveness of lenalidomide in the patients with del 5q-MDS were evaluated.

As per the Japanese Pharmaceutical and Medical Devices Agency Act (PMDA), this PMS study was conducted in accordance with the Good Post-marketing Study Practice (GPSP) guidelines, which do not require written informed consent or institutional review board approval from each participating institution; however, if required by the regulations of the participating medical institutions, informed consent or institutional review board approval was obtained. The protocol was also reviewed and approved by the Ministry of Health, Labour, and Welfare (MHLW) prior to study initiation.

### Study participants and treatment

All individuals who started treatment with lenalidomide for del 5q-MDS after its approval on August 19, 2010 were eligible for inclusion in this analysis. Patients received lenalidomide in 28-day treatment cycles, consisting of oral lenalidomide 10 mg once daily for 21 consecutive days, followed by a 7-day rest period. The lenalidomide dose could be reduced if required by the patient’s condition.

### Study procedures

This study was conducted in two periods: an observation period and a progression to AML period. The observation period followed individuals from the initiation of lenalidomide until the end of six treatment cycles. During the observation period, investigators completed a CRF for each patient on their demographic and clinical characteristics, treatments, outcomes, and adverse drug reactions (ADRs) during the following periods: Cycle 1: from the first day of lenalidomide treatment to immediately before the start of cycle 2; Cycle 2–3: from the start of cycle 2 to immediately before the start of cycle 4; and Cycle 4–6: from the start of cycle 4 to immediately before the start of cycle 7. For individuals who discontinued lenalidomide before six treatment cycles, investigators completed a follow-up CRF that covered from the discontinuation of lenalidomide to when progression to AML was confirmed or 6 months after the start of lenalidomide treatment, whichever occurred first.

In participating institutions, patients with del 5q-MDS without confirmed progression to AML after six treatment cycles could then be included in the progression to AML period, where they were followed until 3 years after starting lenalidomide. CRFs specifically for assessing the progression to AML were separately completed at 6-month intervals, and covered the following periods: from the first day of the seventh cycle of treatment to 1 year after the start of lenalidomide; from 1 year to 1.5 years after the start of lenalidomide; from 1.5 years to 2 years after the start of lenalidomide; from 2 to 2.5 years after the start of lenalidomide; and from 2.5 to 3 years after the start of lenalidomide.

### Outcomes

The safety of lenalidomide in patients with del 5q-MDS was evaluated by recording ADRs, which were defined as any adverse events (AEs) for which a causal relationship with lenalidomide could not be excluded. The number of patients with ADRs and the incidence of each ADR were calculated, and were classified by using the preferred terms (PT) and system organ classes (SOC) in the Japanese version of the Medical Dictionary for Regulatory Activities (MedDRA/J) version 23.0. With regard to ADRs that were considered blood and lymphatic system disorders in MedDRA/J, a reduced platelet count could be classified as either "thrombocytopenia" or "platelet count decreased", a reduced neutrophil count could be classified as either “neutropenia” or “neutrophil count decreased”, a reduced white blood cell count could be classified as either “leukopenia” or “white blood cell count decreased”, and a decrease in hemoglobin could either be classified as “anemia” or “hemoglobin decreased”, all at the discretion of the participating physician. To provide clarity on the real incidence of these ADRs in this study, the number of patients with “thrombocytopenia” and “platelet count decreased”, the number of patients with “neutropenia” and “neutrophil count decreased”, the number of patients with “leukopenia” and “white blood cell count decreased”, and the number of patients with “anemia” and “hemoglobin decreased” were counted as a combined value, respectively. Serious ADRs, ADRs resulting in discontinuation, and ADRs resulting in death were also recorded.

The effectiveness of lenalidomide in patients with del 5q-MDS was evaluated as the proportion of RBC transfusion-dependent patients who achieved RBC transfusion independence. Patients were defined as transfusion dependent if they had received at least one RBC transfusion within ≤ 56 days of the previous infusion in the ≥ 112 days immediately before the start of treatment with lenalidomide. RBC transfusion independence was defined as ongoing clinical status that did not require the administration of RBC transfusions for ≥ 56 consecutive days after starting lenalidomide treatment.

The number of patients who progressed to AML and the time to AML progression was also assessed in both the observational period and the progression to AML period. Furthermore, AML progression was analyzed by IPSS risk category, where the low-risk group included patients in the “low” (IPSS score of 0) and “int-1” (score of 0.5–1.0) risk categories, and the high-risk group included patients in the “int-2” (score of 1.0–1.5) and “high” (score of ≥ 2.5) risk categories.

### Statistical analysis

Based on the results of the MM-009 and MM-010 clinical studies in patients with RRMM [14, 15], an estimated 1000 patients in total would be necessary to collect at least one case of the serious AE “serious rash” (i.e., incidence of 0.3%), which had the lowest incidence among the AEs listed as priority investigation items (excluding teratogenicity), with ≥ 95% probability. Assuming that the study discontinuation rate at 6 months for Japanese patients was the same as that reported in those studies (32.3%) [14, 15], the number of patients to be enrolled was set at 1500 in order to collect data from 1000 patients at 6 months for analysis. As previous studies indicated that there is no significant difference in the safety profile of lenalidomide in patients with RRMM or del 5q-MDS, it was considered possible to also investigate lenalidomide safety in del 5q-MDS based on the number of cases set by RRMM. During the period in which 1500 patients were enrolled, it was estimated that 110 patients with del 5q-MDS would be enrolled, which would give an analysis set of 80 patients, assuming that approximately one-third of patients would discontinue treatment within 6 months.

The safety analysis set included all patients who received at least one dose of lenalidomide. The effectiveness analysis included all patients in the safety analysis set who did not have any of the following protocol deviations: prior lenalidomide treatment; concomitant use of other antineoplastic treatment prior to lenalidomide; or transfer to another hospital.

Summary statistics were used to present patient background characteristics and continuous variables, including the number of patients and proportion for each category, mean ± standard deviation (SD), and median (range). With regard to the calculating date of disease onset, in cases that the day of disease onset was missing, but the month and year were available, it was assumed that the individual developed the disease on the first day of that month. Similarly, in cases that the day and month of disease onset were missing (i.e., only the year of disease onset was available), it was assumed that the individual developed the disease on January 1^st^ of that year. Other missing values were not imputed. The time to AML progression was assessed using Kaplan–Meier methodology.

## Results

### Patient population

**Fig. 1:**
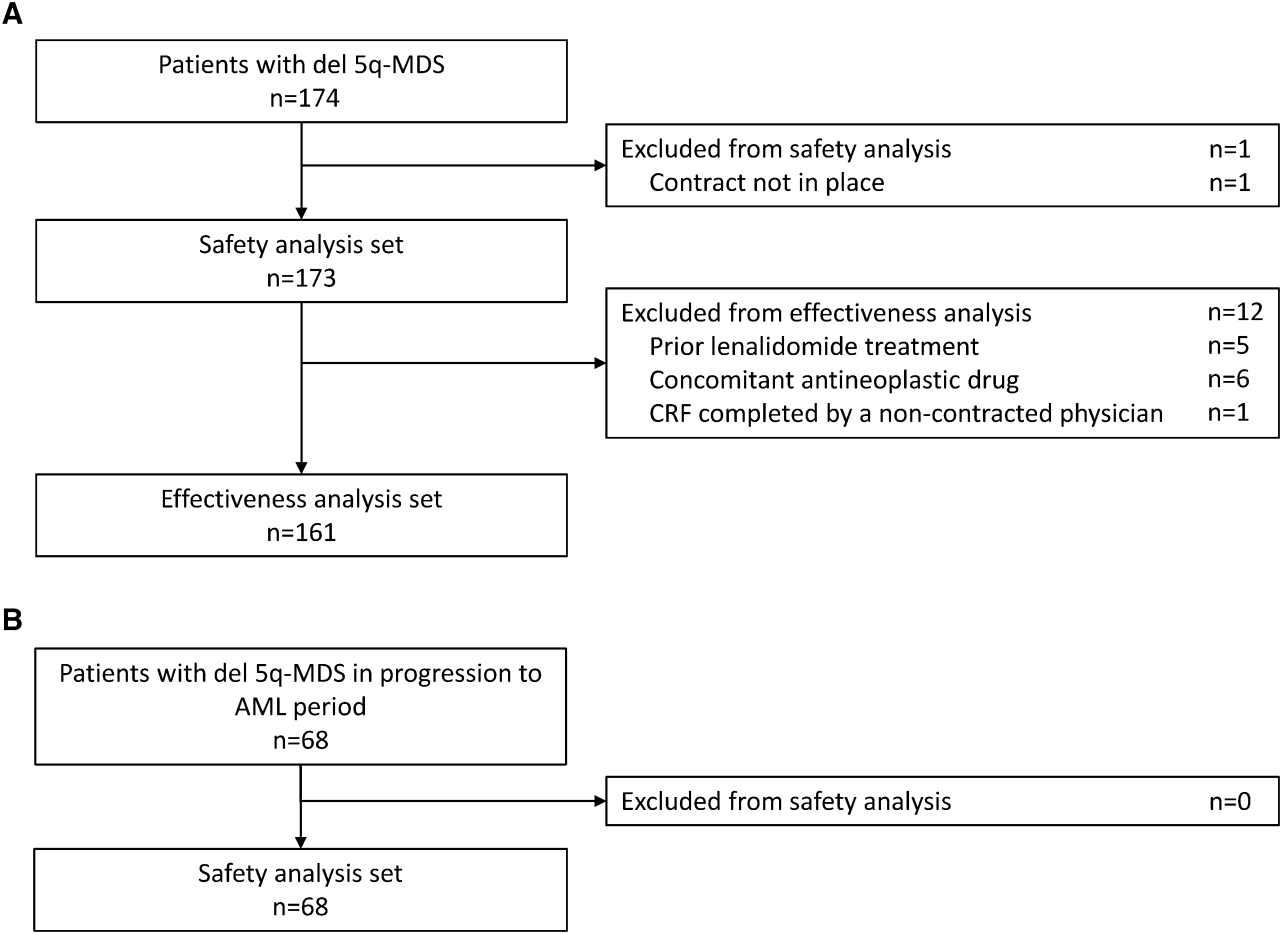
Patient disposition in the **A** observation period and **B** progression to AML period. AML, acute myeloid leukemia; CRF, case report form; del 5q, deletion 5q; MDS, myelodysplastic syndromes

In total, 174 patients with del 5q-MDS started lenalidomide treatment between August 2010 and September 2011 (Fig. 1). One patient was excluded as the survey contract was not in place; therefore, 173 patients with del 5q-MDS were included in the current analysis, and comprised the safety analysis set. In the safety analysis set, 115/173 patients with del 5q-MDS discontinued lenalidomide (66.5%); the most common reason for discontinuation was an AE (25.4%) (Supplementary Table S1, Supplementary Fig. S1). The effectiveness analysis set included 161 of the 173 patients; 12 patients were excluded due to prior lenalidomide (*n* = 5), concomitant antineoplastic therapy (*n* = 6), or a non-contracted physician completing the patient’s CRF (*n* = 1).

Sixty-eight patients enrolled in the progression to AML period and had CRFs collected between February 24, 2011 and June 18, 2020. Of these, 27 patients discontinued lenalidomide (39.7%); the most common reason for discontinuation was progression of underlying disease (22.1%) (Supplementary Table S1; Supplementary Fig. S1).

Patient baseline demographics and clinical characteristics for patients with del 5q-MDS enrolled in the observational and progression to AML periods are summarized in Table 1. In patients enrolled in the observational period, the mean ± SD age was 72.4 ± 9.0 years; the majority of patients (119/173; 68.8%) were aged > 70 years. One hundred and thirteen patients (65.3%) had a disease duration of either < 1 year or ≥ 1 to < 3 years. The most common type of MDS was RA (68/173; 39.3%) by FAB classification and was MDS associated with isolated del 5q (46/173; 26.6%) by WHO 2008 classification. The majority of patients (126/173; 72.8%) had received a prior RBC transfusion, and 114 patients (65.9%) had transfusion-dependent anemia.

**Table 1:** Baseline demographics and patient characteristics in patients with myelodysplastic syndromes with deletion 5q enrolled in the observation and progression to AML periods

	Observation period (*n* = 173)	Progression to AML period^a^ (*n* = 68)
Sex, *n* (%)		
Male	85 (49.1)	29 (42.6)
Female	88 (50.9)	39 (57.4)
Age, years		
Mean ± SD	72.4 ± 9.0	72.6 ± 9.4
Median (range)	74.0 (42.0–88.0)	74.0 (42.0–88.0)
Age category, *n* (%)		
> 40 to 50 years	3 (1.7)	2 (2.9)
> 50 to 60 years	10 (5.8)	4 (5.9)
> 60 to 70 years	41 (23.7)	12 (17.6)
> 70 to 80 years	79 (45.7)	35 (51.5)
> 80 to 90 years	40 (23.1)	15 (22.1)
Hospitalization status, *n* (%)		
Inpatient	71 (41.0)	21 (30.9)
Outpatient	66 (38.2)	36 (52.9)
Inpatient/outpatient	36 (20.8)	11 (16.2)
Disease duration, years	*n* = 154	*n* = 59
Mean ± SD	2.0 ± 2.2	2.9 ± 2.4
Median (range)	1.2 (0.0–13.0)	2.8 (0.1–9.0)
Disease duration category, *n* (%)		
< 1 year	72 (41.6)	19 (27.9)
≥ 1 to < 3 years	41 (23.7)	13 (19.1)
≥ 3 to < 5 years	24 (13.9)	14 (20.6)
≥ 5 to < 10 years	16 (9.2)	13 (19.1)
≥ 10 years	1 (0.6)	0
Unknown/not specified	19 (11.0)	9 (13.2)
ECOG PS, *n* (%)		
0	69 (39.9)	33 (48.5)
1	67 (38.7)	28 (41.2)
2	24 (13.9)	7 (10.3)
≥ 3	13 (7.5)	0
IPSS risk classification, *n* (%)^b^		
Low/int-1	124 (71.7)	58 (85.3)
Int-2/high	48 (27.7)	10 (14.7)
Unknown/not specified	1 (0.6)	–
FAB classification of MDS, *n* (%)		
RA	68 (39.3)	34 (50.0)
RARS	4 (2.3)	1 (1.5)
RAEB	31 (17.9)	7 (10.3)
RAEB-t	12 (6.9)	1 (1.5)
CMML	1 (0.6)	0
Unknown/not specified	57 (32.9)	25 (36.8)
WHO 2008 classification of MDS, *n* (%)		
RCUD	6 (3.5)	2 (2.9)
RARS	3 (1.7)	1 (1.5)
RCMD	21 (12.1)	9 (13.2)
RAEB-1	19 (11.0)	8 (11.8)
RAEB-2	27 (15.6)	3 (4.4)
MDS-U	2 (1.2)	0
MDS with isolated del 5q	46 (26.6)	28 (41.2)
Unknown/not specified	49 (28.3)	17 (25.0)
Bone blast volume, %		
< 5%	100 (57.8)	48 (70.6)
≥ 5 to < 20%	52 (30.1)	14 (20.6)
≥ 20%	6 (3.5)	1 (1.5)
Unknown/not specified	15 (8.7)	5 (7.4)
Prior RBC transfusion, *n* (%)		
No	39 (22.5)	17 (25.0)
Yes	126 (72.8)	45 (66.2)
Unknown/not specified	8 (4.6)	6 (8.8)
RBC transfusion-dependent anemia, *n* (%)	114 (65.9)	43 (63.2)
Presence of complications, *n* (%)	130 (75.1)	49 (72.1)
Hepatic impairment	16 (9.2)	7 (10.3)
Renal impairment	34 (19.7)	12 (17.6)
Peripheral neuropathy	5 (2.9)	3 (4.4)
Infections	12 (6.9)	1 (1.5)
Hypertension	55 (31.8)	21 (30.9)
Hyperlipidemia	14 (8.1)	5 (7.4)
Diabetes mellitus	35 (20.2)	13 (19.1)
Heart disease	35 (20.2)	13 (19.1)
Other	86 (49.7)	31 (45.6)
Prior lenalidomide treatment, *n* (%)	5 (2.9)	2 (2.9)
Concomitant use of antineoplastic drugs, *n* (%)	12 (6.9)	2 (2.9)
Concomitant use of other drugs, *n* (%)	160 (92.5)	64 (94.1)

^a^At the end of the observation period, patients with del 5q-MDS without confirmed progression to AML could be enrolled in the progression to AML period

^b^IPSS risk categories are as follows: low-risk group included patients in the “low” (IPSS score of 0) and “int-1” (score of 0.5–1.0) risk categories, and the high-risk group included patients in the “int-2” (score of 1.0–1.5) and “high” (score of ≥ 2.5) risk categories

AML, acute myeloid leukemia; CMML, chronic myelomonocytic leukemia; del 5q, deletion 5q; ECOG PS, Eastern Cooperative Oncology Group performance status; FAB, French–American–British; IPSS, International Prognostic Scoring System; MDS, myelodysplastic syndromes; MDS-U, myelodysplastic syndromes, unclassifiable; RA, refractory anemia; RAEB, refractory anemia with excess blasts; RAEB-1, RAEB with 5–9% blasts and no Auer rods; RAEB-2, RAEB with 10–19% blasts ± Auer rods; RAEB-t, refractory anemia with excess blasts in transformation; RARS, refractory anemia with ringed sideroblasts; RBC, red blood cell; RCMD, refractory cytopenia with multilineage dysplasia; RCUD, refractory cytopenia with unilineage dysplasia; SD, standard deviation; WHO, World Health Organization

### Lenalidomide exposure

Among patients with del 5q-MDS in the safety analysis set, the mean ± SD total treatment duration (defined as the number of days from the first to the last dose of lenalidomide + 1) was 102.0 ± 97.4 days, with a mean ± SD of 56.7 ± 42.4 actual dosing days (Table 2). The mean ± SD daily dose of lenalidomide was 7.4 ± 2.3 mg.

**Table 2:** Dosing of lenalidomide in patients with myelodysplastic syndromes with deletion 5q (safety analysis set)

	*n* = 173
Total treatment duration (including interruptions)^a^	
Mean ± SD, days	102.0 ± 97.4
Median (range), days	80.0 (3.0–755.0)
Category, *n* (%)	
≤ 28 days	52 (30.1)
29–56 days	20 (11.6)
57–84 days	17 (9.8)
85–112 days	12 (6.9)
113–140 days	13 (7.5)
141–168 days	29 (16.8)
169–365 days	28 (16.2)
≥ 366 days	2 (1.2)
Total treatment duration (actual dosing days)^b^	
Mean ± SD, days	56.7 ± 42.4
Median (range), days	43.0 (3.0–168.0)
Category, *n* (%)	
≤ 21 days	38 (22.0)
21 days	16 (9.2)
22–63 days	59 (34.1)
64–126 days	52 (30.1)
≥ 127 days	8 (4.6)
Daily dose (actual dosing days), mg^c^	
Mean ± SD	7.4 ± 2.3
Median (range)	7.1 (3.6–10.0)

^a^Number of days from date of first to last dose of lenalidomide (+ 1)

^b^Number of days with actual lenalidomide dosing

^c^Calculated as mean ± SD or median (range) of days with actual lenalidomide dosing

SD, standard deviation

### Safety analysis

At least one ADR of any grade occurred in 135/173 patients (78.0%) with del 5q-MDS (Table 3). The most common ADRs by SOC were blood and lymphatic system disorders (64; 37.0%), investigations (62; 35.8%), and skin and subcutaneous tissue disorders (61; 35.3%). The most common ADRs (occurring in ≥ 5% of patients) by MedDRA/J PT were thrombocytopenia or platelet count decreased (80; 46.2%), neutropenia or neutrophil count decreased (73; 42.2%), rash (40; 23.1%), white blood cell count decreased (14; 8.1%), anemia or hemoglobin decreased (12; 6.9%), and pneumonia (9; 5.2%) (Supplementary Table S2).

**Table 3:** Summary of adverse drug reactions in patients with myelodysplastic syndromes with deletion 5q (safety analysis set; *n* = 173)

ADR, *n* (%)^a^	Any	Serious
At least one ADR	135 (78.0)	88 (50.9)
Blood and lymphatic system disorders	64 (37.0)	45 (26.0)
Investigations	62 (35.8)	37 (21.4)
Skin and subcutaneous tissue disorders	61 (35.3)	8 (4.6)
Infections and infestations	21 (12.1)	15 (8.7)
Gastrointestinal disorders	18 (10.4)	3 (1.7)
General disorders and administration site conditions	11 (6.4)	1 (0.6)
Cardiac disorders	6 (3.5)	6 (3.5)
Hepatobiliary disorders	5 (2.9)	2 (1.2)
Nervous system disorders	5 (2.9)	3 (1.7)
Vascular disorders	5 (2.9)	4 (2.3)
Neoplasms benign, malignant and unspecified (including cysts and polyps)^b^	3 (1.7)	3 (1.7)
Metabolism and nutrition disorders	3 (1.7)	1 (0.6)
Renal and urinary disorders	3 (1.7)	2 (1.2)
Respiratory, thoracic and mediastinal disorders	3 (1.7)	2 (1.2)
Ear and labyrinth disorders	2 (1.2)	2 (1.2)
Endocrine disorders	2 (1.2)	0
Eye disorders	1 (0.6)	0
Immune system disorders	1 (0.6)	1 (0.6)
Psychiatric disorders	1 (0.6)	0

^a^System organ class according to Medical Dictionary for Regulatory Activities for Japan

^b^All reported ADRs were progression to acute myeloid leukemia. Reporting of progression to acute myeloid leukemia as an ADR was at the discretion of the participating physician, and as such, of the 19 patients who experienced acute myeloid leukemia progression in this study, only 3 were reported to have this ADR

ADR, adverse drug reaction

Serious ADRs were reported in 88/173 patients (50.9%). The most common serious ADRs by SOC were blood and lymphatic system disorders (45; 26.0%), investigations (37; 21.4%), infections or infestations (15; 8.7%), and skin and subcutaneous tissue disorders (8; 4.6%) (Table 3). The most common serious ADRs by PT (occurring in ≥ 2% of patients) were neutropenia or neutrophil count decreased (54; 31.2%), thrombocytopenia or platelet count decreased (45; 26.0%), anemia or hemoglobin decreased (10; 5.8%), pneumonia (8; 4.6%), white blood cell count decreased (7; 4.0%), cardiac failure (6; 3.5%), and rash (4; 2.3%) (Supplementary Table S2).

The profile of Grade ≥ 3 events was similar to the profile of serious ADRs, with the most common (occurring in ≥ 2% of patients) being neutropenia or neutrophil count decreased (42/173; 24.3%), thrombocytopenia or platelet count decreased (26; 15.0%), pneumonia (7; 4.0%), white blood cell count decreased (7; 4.0%), anemia or hemoglobin decreased (5; 2.9%) and cardiac failure (4; 2.3%). Some patients with del 5q-MDS developed an ADR that was not listed in the Precautions section of the prescribing information. These events were AML and transformation of MDS (*n* = 3 each; 1.7%) and gas gangrene, pneumonia, sepsis, anemia, hemolytic anemia, neutropenia, thrombocytopenia, hemorrhagic cerebral infarction, syncope, sensorineural deafness, sudden deafness, cardiac failure, pericardial effusion, thrombophlebitis, anal fissure, toothache, asteatotic eczema, nephrotic syndrome, pollakiuria, multiple organ dysfunction syndrome, thyroid function test abnormal, and blood immunoglobulin E increased (*n* = 1 each; 0.6%).

Eleven ADRs leading to death were reported in six patients and included gas gangrene, pneumonia, sepsis, acute myeloid leukemia, transformation of MDS, anemia, thrombocytopenia, neutropenia, hemorrhagic cerebral infarction, heart failure and multiple organ dysfunction syndrome. None of the fatal ADRs had a clear relationship to administration of lenalidomide, so none of the ADRs resulting in death required new safety measures.

When assessed by IPSS risk category, at least one ADR of any grade occurred in 104/124 (83.9%) patients in the low/int-1 risk category and in 30/48 (62.5%) patients in the int-2/high risk category (Supplementary Table S3).

### Effectiveness analysis

Among the 161 patients in the effectiveness analysis set, 114 (70.8%) were RBC transfusion dependent at baseline (Table 4). Of these, 39/114 (34.2%) became transfusion independent during lenalidomide treatment.

**Table 4:** Red blood cell transfusion-dependent anemia (effectiveness analysis set)

	*n* = 161
RBC transfusion-dependent anemia at treatment initiation, *n* (%)	
No	47 (29.2)
Yes	114 (70.8)
RBC transfusion independence during treatment, *n* (%)	*n* = 114
No	75 (65.8)
Yes	39 (34.2)

RBC, red blood cell

When assessed by IPSS risk category, 35/80 (43.7%) in the low/int-1 risk category and 4/34 (11.8%) in the int-2/high risk category achieved transfusion independence.

### Progression to AML

During the observation period, 19 patients (11.0%) among 173 patients developed AML (Table 5). When assessed by IPSS risk category, 8/124 (6.5%) patients in the low/int-1 risk category and 11/48 (22.9%) in the int-2/high risk category developed AML. The mean ± SD time from starting lenalidomide therapy to AML progression in the observational period was 102.8 ± 69.1 days in the low/int-1 risk category and 56.0 ± 51.2 days in the int-2/high risk category.

**Table 5:** Proportion of patients who developed acute myeloid leukemia in the observational period overall, and by International Prognostic Scoring System risk category

	Overall (*n* = 173)	Low/int-1 (*n* = 124)^a^	Int-2/high (*n* = 48)^b^
Progression to AML, *n* (%)			
No	154 (89.0)	116 (93.5)	37 (77.1)
Yes	19 (11.0)	8 (6.5)	11 (22.9)
Time to confirmation of progression, days	*n* = 18^c^	*n* = 8	*n* = 10^c^
Mean ± SD	76.8 ± 62.7	102.8 ± 69.1	56.0 ± 51.2
Median (range)	54.5 (14.0–210.0)	66.0 (44.0–210.0)	24.0 (14.0–136.0)
Category, *n* (%)			
< 21 days	2 (1.2)	0	2 (4.2)
21 days	1 (0.6)	0	1 (2.1)
22–63 days	8 (4.6)	4 (3.2)	4 (8.3)
64–126 days	2 (1.2)	1 (0.8)	1 (2.1)
127–252 days	5 (2.9)	3 (2.4)	2 (4.2)
253–378 days	0	0	0
379–504 days	0	0	0
505–630 days	0	0	0
631–756 days	0	0	0
≥ 757 days	0	0	0

^a^The low-risk group included patients in the “low” (IPSS score of 0) and “int-1” (score of 0.5–1.0) risk categories

^b^The high-risk group included patients in the “int-2” (score of 1.0–1.5) and “high” (score of ≥ 2.5) risk categories

^c^Time to confirmation of progression was not reported in one patient

AML, acute myeloid leukemia; IPSS, International Prognostic Scoring System; SD, standard deviation

During the progression to AML period, 12 patients (17.6%) among 68 patients developed AML (Table 6). When assessed by IPSS risk category, 10/58 (17.2%) patients developed AML in the low/int-1 risk category and 2/10 (20.0%) in the int-2/high risk category. The mean ± SD time from starting lenalidomide therapy to AML progression in the progression to AML period was 753.1 ± 202.6 days in the low/int-1 risk category and 453.0 ± 28.3 days in the int-2/high risk category.

**Table 6:** Proportion of patients who developed acute myeloid leukemia in the progression to AML period^a^ overall, and by International Prognostic Scoring System risk category

	Overall (*n* = 68)	Low/int-1 (*n* = 58)^b^	Int-2/high (*n* = 10)^c^
Progression to AML, *n* (%)			
No	56 (82.4)	48 (82.8)	8 (80.0)
Yes	12 (17.6)	10 (17.2)	2 (20.0)
Time to confirmation of progression, days	*n* = 12	*n* = 10	*n* = 2
Mean ± SD	703.1 ± 217.5	753.1 ± 202.6	453.0 ± 28.3
Median (range)	671.5 (340.0–1013.0)	721.0 (340.0–1013.0)	453.0 (433.0–473.0)
Category, *n* (%)			
127–252 days	0	0	0
253–378 days	1 (1.5)	1 (1.7)	0
379–504 days	2 (2.9)	0	2 (20.0)
505–630 days	0	0	0
631–756 days	4 (5.9)	4 (6.9)	0
≥ 757 days	5 (7.4)	5 (8.6)	0

^a^At the end of the observation period, patients with del 5q-MDS without confirmed progression to AML could be enrolled in the progression to AML period

^b^The low-risk group included patients in the “low” (IPSS score of 0) and “int-1” (score of 0.5–1.0) risk categories

^c^The high-risk group included patients in the “int-2” (score of 1.0–1.5) and “high” (score of ≥ 2.5) risk categories

AML, acute myeloid leukemia; del 5q, deletion 5q; IPSS, International Prognostic Scoring System; MDS, myelodysplastic syndromes; SD, standard deviation

## Discussion

Our PMS study in this real-world population of Japanese patients with del 5q-MDS who received lenalidomide confirm the results of randomized clinical trials on its safety, efficacy, and the likelihood of AML progression in this patient population.

The most common ADRs in our study were similar to those in the clinical trials and no new safety concerns were identified in Japanese patients [12, 13]. At the time of lenalidomide approval, two clinical trials had been conducted in Japanese patients (MDS-007 and MM-017), and in both studies, close to 100% of patients developed an AE [13, 16]. The overall ADR incidence in this real-world study was a little lower than the incidence of AEs in previous studies (78% in del 5q-MDS patients), but the type of AEs, including Grade ≥ 3 AEs, seen in those studies was similar to the type of ADRs and Grade ≥ 3 ADRs observed in our analysis, with hematologic AEs predominating [13, 16]. Approximately half of the patients with del 5q-MDS in our study developed a serious ADR, whereas no serious AEs were reported in the phase 2 MDS-007 study [13]. This likely reflects the heterogeneous patient population of our study, which included all patients receiving lenalidomide, including those with higher risk disease (27.7% of patients in our study had IPSS int-2/high-risk disease compared with none in the MDS-007 study) [13]. The MDS-007 study also excluded patients with low platelet or neutrophil counts at baseline or with impaired renal/hepatic function [13], whereas no such limits were placed on the inclusion of patients in this PMS study. Our patient cohort also included a higher proportion of patients aged > 70 years (68.8% compared with 55% in MDS-007 [13]), which may indicate a population more vulnerable to ADRs, as there are data indicating that older patients are more likely than younger ones to require lenalidomide dose adjustments due to AEs, especially if the patients have comorbidities [17, 18].

Among the 114 patients who were transfusion dependent at baseline in our study, 39 (34.2%) achieved transfusion independence. This is lower than the rate in the Japanese phase 2 clinical study, in which 5/5 (100%) patients achieved transfusion independence [13]. It is also lower than the transfusion-independence rate seen in the international MDS-004 study, although in that study, the transfusion-independence rate ranged from 56.1% (23 of 41 patients) [95% confidence interval (CI) 34.9, 65.1] to 61.0% (25 of 41 patients) [95% CI 44.5, 75.8] [12], which is closer to the result obtained in our study. Although it is difficult to directly compare transfusion-independence rates between these controlled studies and our surveillance study of all-comers, the likely reason for the difference in findings is that patients in our study were more heterogeneous than the patients in the MDS-004 or MDS-007 studies, and included 6.9% of patients with a FAB classification of RAEB-t and 27.7% in the IPSS risk category of int-2 or high. Indeed, a previous study in patients with int-2-/high-risk del 5q-MDS reported a transfusion-independence rate of 25%, and an even lower rate in patients with other chromosomal abnormalities in addition to del 5q [19]. In this study, the transfusion independence rate in patients with the IPSS risk category of int-2 or high was lower than that in patients with the IPSS risk category of low or int-1.

Our study in Japanese patients with del 5q-MDS found that 6.5% of patients in the low-/int-1-risk category and 22.9% of those in the int-2-/high-risk category developed AML after lenalidomide treatment during the observational period and 17.2% and 20.0%, respectively, during the progression to AML period. These rates are consistent with an analysis of AML progression in 381 low-/int-1-risk untreated patients with del 5q-MDS reported by Germing and colleagues in 2012 [20]. In that study, 9% of patients had progressed to AML at 2 years and 25% at 5 years. Therefore, the AML-progression rate over 3 years among patients in the same risk categories in our study was consistent with previous findings, and suggests that lenalidomide does not increase the risk of AML progression.

The limitations of our PMS study are typical of those for an observational analysis, including no control group and a heterogeneous patient population. However, the latter is also a strength, because the findings are generalizable to the real-world population of del 5q-MDS patients seen in routine clinical practice in Japan. In addition, as there was no central review of outcomes, we were reliant on the accuracy of reporting by investigators. Finally, the safety and effectiveness of lenalidomide was only assessed until the end of six treatment cycles, while the progression to AML was assessed until 3 years. As such, the safety and transition to AML beyond this period are unknown and this is another limitation of this study.

In conclusion, lenalidomide treatment of patients with del 5q-MDS did not cause any new safety concerns in routine clinical use in Japan. Furthermore, we found no evidence for an increased risk of AML progression among patients who received lenalidomide.

### Supplementary Information

Below is the link to the electronic supplementary material.

Supplementary file1 (DOCX 96 KB)

## Data Availability

BMS policy on data sharing may be found at https://www.bms.com/researchers-and-partners/clinical-trials-and-research/disclosure-commitment.html.
