# Supplementary material for: Lenalidomide treatment of Japanese patients with myelodysplastic syndromes with 5q deletion: a post-marketing surveillance study
Source: Int J Hematol. 2023 Jul 26;118(4):432–42. doi: 10.1007/s12185-023-03634-7 (PMC10522743; doi:10.1007/s12185-023-03634-7)

**Lenalidomide treatment of Japanese patients with myelodysplastic syndromes with 5q deletion: a post-marketing surveillance study**

**Supplementary Materials**

**Authors:** Shuji Uno^1^, Yoko Motegi^2^, Kenichi Minehata^1^, Yasuo Aoki^2^

**Affiliations:** ^1^Japan Medical-Hematology, Bristol-Myers Squibb K.K., Tokyo, Japan; ^2^PMS Operations, Worldwide Patient Safety Japan, Bristol-Myers Squibb K.K., Tokyo, Japan.

**Corresponding author:** Shuji Uno, Japan Medical-Hematology, Bristol-Myers Squibb K.K., Otemachi One Tower, 1-2-1 Otemachi, Chiyoda-ku, Tokyo, 100-0004, Japan. TEL: +81-3-6706-1404; FAX: +81-3-6705-7929; Email: [shuji.uno@bms.com](mailto:shuji.uno@bms.com)

## Supplementary Table S1.

Discontinuation of lenalidomide treatment in the observation and progression to AML periods

|  | **Observation period**  **(*n* = 173)** | **Progression to AML period**  **(*n* = 68)^a^** |
| --- | --- | --- |
| Total discontinuations, *n* (%) | 115 (66.5) | 27 (39.7) |
| Reasons for discontinuation, *n* (%) |  |  |
| AE | 44 (25.4) | 3 (4.4) |
| Progression of underlying disease | 40 (23.1) | 15 (22.1) |
| Patient request (other than an AE) | 10 (5.8) | 5 (7.4) |
| Change of hospital | 5 (2.9) | 0 |
| Death | 4 (2.3) | 0 |
| Other | 12 (6.9) | 4 (5.9) |

^a^At the end of the observation period, patients with del 5q-MDS without confirmed progression to AML could be enrolled in the progression to AML period.

AE, adverse event; AML, acute myeloid leukemia; del 5q, deletion 5q; MDS, myelodysplastic syndromes.

## Supplementary Table S2.

Summary of adverse drug reactions occurring in ≥5% or serious adverse drug reactions occurring in ≥2% of patients with myelodysplastic syndromes with deletion 5q (safety analysis set; *n* = 173)

| **ADR, *n* (%)^a^** | **Any** | **Serious** |
| --- | --- | --- |
| Blood and lymphatic system disorders | 64 (37.0) | 45 (26.0) |
| Neutropenia | 43 (24.9) | 31 (17.9) |
| Thrombocytopenia | 42 (24.3) | 23 (13.3) |
| Anemia | 7 (4.0) | 6 (3.5) |
| Investigations | 62 (35.8) | 37 (21.4) |
| Platelet count decreased | 38 (22.0) | 22 (12.7) |
| Neutrophil count decreased | 30 (17.3) | 23 (13.3) |
| White blood cell count decreased | 14 (8.1) | 7 (4.0) |
| Hemoglobin decreased | 5 (2.9) | 4 (2.3) |
| Infections and infestations | 21 (12.1) | 15 (8.7) |
| Pneumonia | 9 (5.2) | 8 (4.6) |
| Skin and subcutaneous tissue disorders | 61 (35.3) | 8 (4.6) |
| Rash | 40 (23.1) | 4 (2.3) |
| Cardiac disorders | 6 (3.5) | 6 (3.5) |
| Cardiac failure | 6 (3.5) | 6 (3.5) |

^a^Preferred term according to Medical Dictionary for Regulatory Activities for Japan.

ADR, adverse drug reaction.

## Supplementary Table S3.

Summary of adverse drug in patients with myelodysplastic syndromes with deletion 5q reactions by International Prognostic Scoring System risk category (safety analysis set; *n* = 173^a^)

| **ADR, *n* (%)^b^** | **Low/int-1 (*n* = 124)** | **Int-2/high (*n* = 48)** |
| --- | --- | --- |
| At least one ADR | 104 (83.9) | 30 (62.5) |
| Blood and lymphatic system disorders | 52 (41.9) | 12 (25.0) |
| Skin and subcutaneous tissue disorders | 51 (41.1) | 9 (18.8) |
| Investigations | 49 (39.5) | 12 (25.0) |
| Gastrointestinal disorders | 14 (11.3) | 3 (6.3) |
| Infections and infestations | 12 (9.7) | 9 (18.8) |
| General disorders and administration site conditions | 9 (7.3) | 2 (4.2) |
| Hepatobiliary disorders | 5 (4.0) | 0 |
| Nervous system disorders | 5 (4.0) | 0 |
| Vascular disorders | 5 (4.0) | 0 |
| Cardiac disorders | 3 (2.4) | 3 (6.3) |
| Respiratory, thoracic and mediastinal disorders | 3 (2.4) | 0 |
| Metabolism and nutrition disorders | 2 (1.6) | 1 (2.1) |
| Renal and urinary disorders | 2 (1.6) | 1 (2.1) |
| Endocrine disorders | 2 (1.6) | 0 |
| Ear and labyrinth disorders | 1 (0.8) | 1 (2.1) |
| Eye disorders | 1 (0.8) | 0 |
| Psychiatric disorders | 1 (0.8) | 0 |
| Neoplasms benign, malignant and unspecified (including cysts and polyps) | 0 | 3 (6.3) |
| Immune system disorders | 0 | 1 (2.1) |

^a^The International Prognostic Scoring System risk category was unknown for one patient.

^b^System organ class according to Medical Dictionary for Regulatory Activities for Japan.

ADR, adverse drug reaction.

## Supplementary Fig. S1.

Patient disposition in the (A) observation period and (B) the progression to AML period and reasons for study withdrawal.
^a^Other than an AE.
AE, adverse event; AML, acute myeloid leukemia; CRF, case report form.


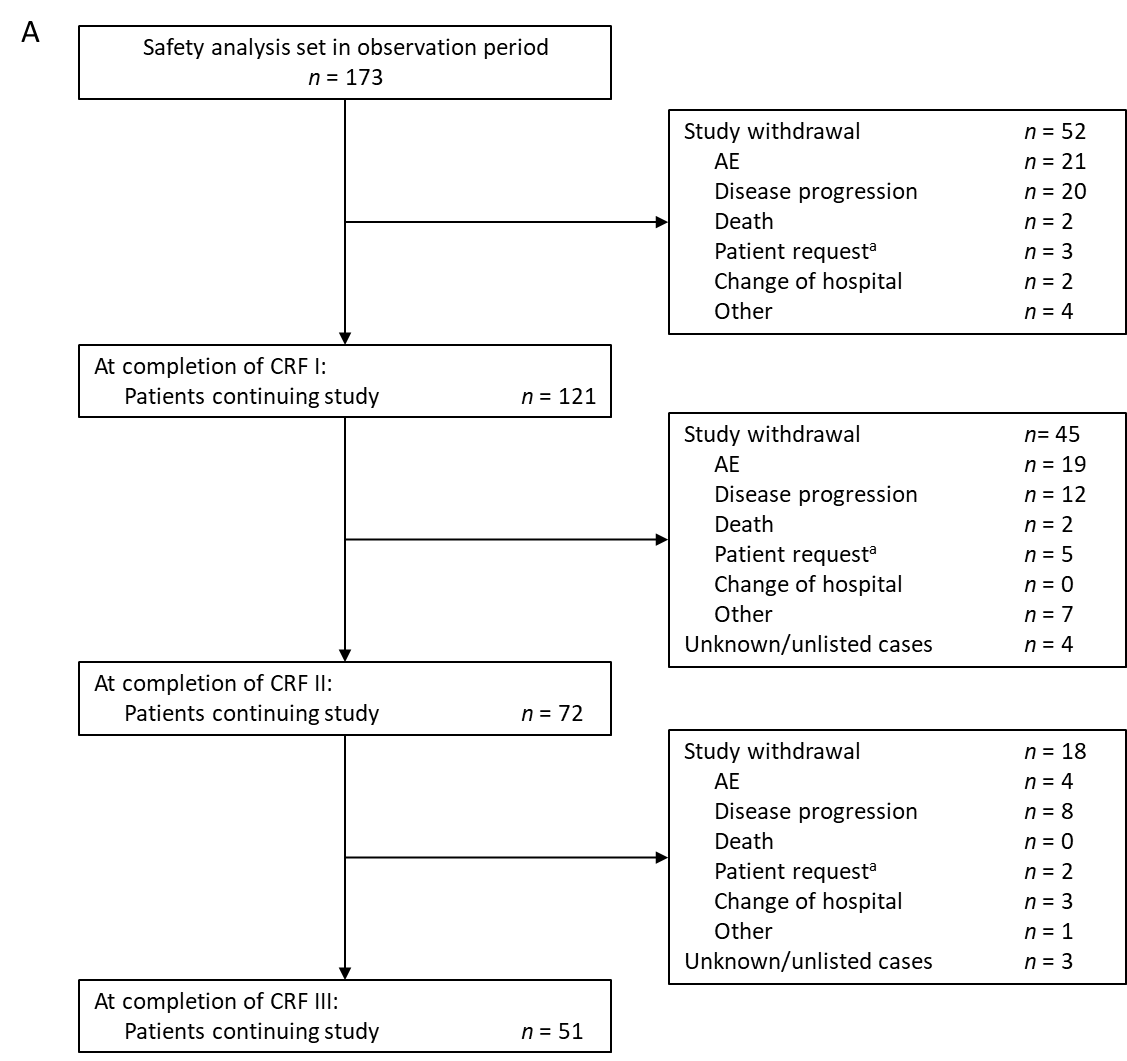


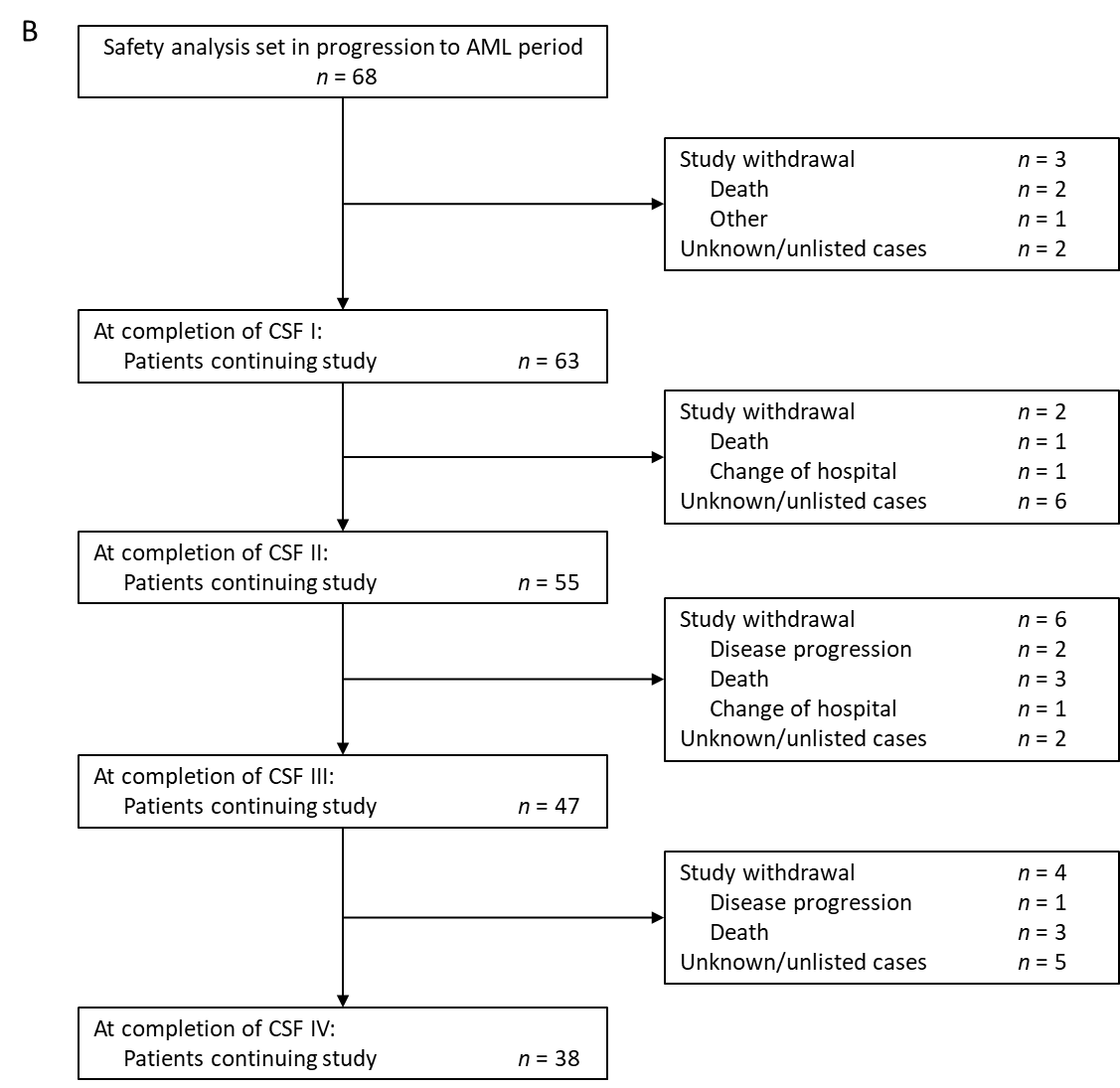

Supplement: Supplementary file 1 — Supplementary file1 (DOCX 96 KB) [file 12185_2023_3634_MOESM1_ESM.docx]
